# Validation of a noninvasive technique to quantify stress in northern bobwhite (*Colinus virginianus*)

**DOI:** 10.1093/conphys/coaa026

**Published:** 2020-04-13

**Authors:** Jessica L Mohlman, Kristen J Navara, Michael J Sheriff, Theron M Terhune, James A Martin

**Affiliations:** 1 D. B. Warnell School of Forestry and Natural Resources, University of Georgia, Athens, GA, 30602, USA; 2 Department of Poultry Science, University of Georgia, Athens, GA 30602, USA; 3 Biology Department, University of Massachusetts Dartmouth, Dartmouth, MA, 02747, USA; 4 Tall Timbers, Tallahassee, FL 32312, USA; 5 Savannah River Ecology Lab, University of Georgia, Athens, GA, 30602, USA

**Keywords:** Northern Bobwhite, validation, stress, fecal corticosterone metabolites, ACTH challenge, biological stressor

## Abstract

Examination of the endocrine system through non-invasive fecal sampling may improve population management more than using demographic indicators alone. By addressing the physiological mechanisms that are influencing fitness, management actions can be proactively developed to alleviate stressors. Proactive determination of vulnerable populations is critical for species of concern, such as the Northern Bobwhite (*Colinus virginianus*), which have suffered decades of population decline. We validated an assay to noninvasively measure the adrenocortical response of captive reared bobwhite through fecal corticosterone metabolites (FCM). All individuals received three sequential 48-hour treatments in which samples were collected every 4 hours, including a reference period, an adrenocorticotropic hormone (ACTH) challenge and a biological stressor (exposure to a hunting dog). Reference FCM values had a mean concentration of 16.75 pg/mg (95% CrI: 13.68, 19.91) with adrenocortical activity increasing by 73% for the duration of the ACTH challenge (29.00 pg/mg; CrI: 25.01, 33.78). FCM concentrations remained similar to that of the reference levels during the biological stressor (16.56 pg/mg; CrI: 13.33, 19.92). Our study validates the use of feces to detect changes in FCM levels in our subject species but also demonstrates the complexity of FCM and the importance of both physiological and biological validation prior to field implementation.

## Introduction

Northern Bobwhite (*Colinus virginianus*; hereafter bobwhite) have experienced considerable population declines throughout their geographic range since the 1920s (Stoddard, 1931; Brennan, 1991; Hernández *et al.*, 2013). Between the years of 1969 and 2004, it was estimated that bobwhite populations decreased by 3.56% (95% CI = [−3.80%, −3.32%]) per year (Link *et al*., 2008). Broad scale changes in land use, including modern agricultural practices, reductions in prescribed fire and changes in forestry practices have resulted in habitat loss that is the primary cause of this decline (Brennan, 1991; Guthery *et al*., 2000; Williams *et al.*, 2004). Bobwhite popularity as a game species in conjunction with their decline has made the species a conservation priority and their designation as a flagship species for many upland and grassland bird communities (Brennan and Kuvlesky, 2005; Crosby *et al*., 2015).

Scientists and managers often estimate bobwhite vital rates (e.g. survival and fecundity) at patch and landscape scales as a way of evaluating the average fitness of individuals in a specific population (Seckinger *et al*., 2008; Riddle *et al*., 2010). Although estimates of vital rates are important, the underlying mechanisms including physiological processes influencing those rates are often unknown. A well-conserved response across vertebrates, when presented with a perceived threat, is the activation of the hypothalamus–pituitary–adrenal (HPA) axis and resultant secretion of glucocorticoids into the blood stream (Sapolsky *et al*., 2000; McEwen and Wingfield, 2003). These hormones promote processes beneficial to survival such as increases in energy mobilization and metabolic rate and reduce costly processes such as reproduction and immunity (Sheriff *et al*., 2009b; Sapolsky *et al*., 2000). Though beneficial in the short term and necessary for survival, extended chronic elevations of circulating glucocorticoid levels can potentially have negative effects on fitness (Sapolsky *et al*., 2000; Macleod *et al.* 2018). While the relationship between stress-induced hormone levels and fitness is not always constant and present, it does provide an additional manner of assessing a population’s robustness in addition to traditional population survey methods (e.g. abundance estimates) (Breuner *et al*., 2008; Bonier *et al*., 2009). Therefore, measuring stress-induced hormone levels may provide an early detection method to assess population vital rates of managed and at-risk populations.

The ability to make inferences using measures of hormone levels requires validation of stress hormone level estimates and their sensitivity to environmental stressors. The most common way to quantify glucocorticoids is through blood plasma (Harlow *et al.*, 1990; Wingfield *et al*., 1994; Hood *et al*., 1998); however, there are significant limitations to the application of this approach for at-risk and wild populations (Le Maho *et al.*, 1992; Cook *et al*., 2000). First, there is the general limitation of acquiring blood samples on wild free-ranging animals. Second, and more importantly, capturing animals and extraction of blood plasma is problematic due to its invasive nature that may lead to imprecise results (Touma and Palme, 2005; Dantzer *et al.*, 2014). Therefore, the invasiveness of this method makes it challenging to use on wild populations and impractical for populations of conservation concern.

Because of the limitations of estimating plasma concentrations of stress hormones, other noninvasive methods to quantify stress such as fecal sampling, urine, feathers and hair have been increasingly used in recent years (Davis *et al*., 2008; Sheriff *et al*., 2011a). Specifically, fecal sampling has become increasingly popular in both domestic and wild vertebrates in a variety of disciplines (Wasser *et al*., 1997; Möstl and Palme, 2002; Washburn *et al*., 2003; Touma *et al*., 2004; Sheriff *et al.*, 2011b; Dantzer *et al.*, 2014; Sheriff *et al.*, 2017; Shipley *et al.*, 2019). Fecal sampling may be used to quantify stress as glucocorticoids within plasma are largely metabolized by the liver and excreted into the gut via bile ducts (for further information see Sheriff *et al.*, 2011a). Fecal sampling is thought to minimize the problems associated with blood plasma sampling due to the lag time between glucocorticoid spikes and detectable increasing metabolite concentrations in the feces (Wasser *et al*., 2000; Palme, 2005). Additionally, fecal samples reflect an average level of circulating glucocorticoids over a period of time, rather than a point sample, potentially providing a more accurate estimate of more chronic glucocorticoid concentrations (Harper and Austad, 2000; Sheriff *et al.*, 2010; Dantzer *et al.*, 2014).

While the use of fecal sampling is less invasive than plasma, relationships to blood glucocorticoid concentrations can require species-specific validation (Millspaugh and Washburn, 2004; Möstl *et al.*, 2005: Palme, 2018). Validation is critical as enterohepatic circulation causes variation in fecal glucocorticoid metabolite (FGM) profiles as well as the duration of stress-response time among different species (Goymann, 2005). Excretion times also differ among species resulting in varying time intervals in which individuals can be sampled, effecting when variation in stress responses can be detected (Goymann, 2005). These differences are not only species specific but can also be age and sex specific (Touma and Palme, 2005; Dantzer *et al*., 2014) and diet, reproductive status, season and time of day can affect FGM levels (Dantzer *et al*., 2014). Due to the numerous factors that may influence FGM, variability in responses among individuals is expected. Therefore, validation studies must include multiple individuals from varying age and sex classes of a known reproductive status in order to accurately assess FGM levels (Palme, 2005).

Validation demonstrates that a technique and assay are biologically relevant through the detection of biologically meaningful alterations in the endocrine status of individuals (Touma and Palme, 2005). The relevance of an assay is determined through both physiological and biological validation (Touma and Palme, 2005). Physiological validation is accomplished through pharmacologically inducing physiological changes in circulating corticosterone levels to evaluate whether these changes are reflected in measured concentrations of FGM (Touma and Palme, 2005). The most widely used experiment to stimulate adrenocortical activity is an adrenocorticotropic hormone (ACTH) challenge test (Palme, 2005; Touma and Palme, 2005). Evaluation of an individual’s reaction to a known stressful event (e.g. capture, immobilization, transport) is also of great importance as it allows for biological validation of an assay. While it is recognized that biological validation is essential for determining the relevance of an assay technique (Palme, 2005; Touma and Palme, 2005), many studies rely solely on the results of the physiological validation (Baltic *et al.*, 2005; Thiel *et al*., 2005; Rettenbacher *et al.*, 2004). Additionally, many of the studies employing biological validation experiments are not necessarily biologically significant or akin to events individuals would encounter in the wild (e.g. saline injections) (Dehnhard *et al.*, 2003; Washburn *et al.*, 2003).

Within this study we describe the use and validation of a noninvasive, field-deployable method to characterize the endocrine response of bobwhite using fecal samples. Endocrine response was measured using fecal corticosterone metabolites (FCM), as corticosterone is the primary glucocorticoid in avian species (Korte *et al.*, 1997). We hypothesized that the FCM content of feces indicates the activity of the HPA axis in bobwhites. We tested this by comparing the responses of 24 captive reared bobwhite comparing age and sex classes to both a biological and a physiological stimulation of the HPA axis over a 7-day period with sampling occurring every 4 hours. Differences in FCM concentrations between sex and age classes were expected to be minimal, as the study did not occur during the breeding season when levels may differ among groups (Touma and Palme, 2005; Dantzer *et al.*, 2014). Following a habituation period, we collected reference fecal samples and then we pharmaceutically stimulated the HPA axis by injecting ACTH to test whether the FCM content of the feces reflects the activity of the HPA axis (Goymann, 2005; Palme, 2005; Touma and Palme, 2005). The study concluded with the implementation of a biological stressor of an anticipated stressful encounter (i.e. a hunting dog), which was expected to increase secretion of corticosterone levels (Touma and Palme, 2005). We predicted that, if the assay accurately reflects HPA activity in this species, FCM concentrations during the duration of the ACTH treatment and the biological stressor should be elevated compared to concentrations in reference fecal samples (Goymann, 2005; Touma and Palme, 2005).

## Methods

### Study Area

The study was conducted at Tall Timbers Research Station approximately 34 km north of Tallahassee, Florida, within the Red Hills Region. Existing aviaries located on the property were used for the study and were located away from high traffic areas to minimize anthropogenic disturbance. Aviaries (7.4 m × 2.3 m × 1.7 m) were constructed using wood framing, which was buried below the ground to prevent disturbance from snakes or other burrowing animals. Walls and roofs of the aviaries were constructed with poultry netting and shade cloth to minimize anthropogenic disturbance and provide protection from weather events. The ground of each aviary was cleared of vegetation to ensure droppings were easily located. Only investigators directly working on the study were allowed within the facility of the aviary area to minimize additional disturbance on birds. Each aviary enclosed one or two smaller pens (76.2 cm × 76.2 cm × 45.72 cm) that held each bobwhite individually and minimized movement to prevent injury and localize feces. Pens were constructed with coated wiring to reduce chance of injury and shade cloth for weather protection. Investigator access to the pens was located on top to allow for easy sample collection and bird handling. Within each pen individuals were provided sorghum from a feeding trough and water for the duration of the study.

### Experiential Design

Twenty-four F1 captive reared bobwhite (six adult males, six adult females, six juvenile males, six juvenile females) from the same genetic stock were randomly assigned to each individual pen within the aviaries. All individuals were weighed, sexed, aged and given unique number leg bands (National Band & Tag Company) prior to being placed within their uniquely identified pens.

All bobwhites were subjected to a 24-hour habituation period to allow for adjustment to the pens and human interaction, as well as to determine the defecation rate for collection. Defecation rate was determined by initially visiting pens every 2 hours for collection and increased to 4 hours when over 50% of the birds were defecating within that time frame. Four-hour collection periods were used for the remainder of the study after the completion of the habituation period, as it was found to be representative of bobwhite defecation rate with a majority of the birds defecating within the time frame. Following the habituation period, all bobwhites were subjected to three 48-hour sequential treatments. The treatments included a reference collection (no manipulation), an ACTH challenge and a biological stressor.

### Sample Collection

Samples were collected every 4 hours throughout all treatments. This was determined as the defecation rate of bobwhite during the habituation period and also minimized additional stress factors that may have been caused by frequent interactions with the investigators. At each collection time point, all fecal droppings were removed from each pen using sterilized tweezers and were placed in a plastic zip bag with a unique identification card. Each identification card denoted the unique individual leg-band number, aviary, pen, sex, age, treatment, date and time of the sample collected. Within the 4-hour collection time frame, individuals may have defecated multiple times with samples ranging up to potentially 4-hours old. Due to the fact that this study occurred during the winter months, samples did not warm up during this time frame. All samples were kept on ice once collected during collection periods. Samples were stored in an on-site freezer at roughly −23°C during the duration of the study. Samples were then transported on ice back to the University of Georgia for long-term storage in a −80°C freezer.

### Treatments

Individuals received no manipulation during the reference period. Samples collected during the reference treatment provided basal levels of FCM in all individuals to allow for comparison within the latter treatments. Prior to the initiation of the ACTH treatment, ACTH (catalog no. A6303, Sigma-Alrich) dosages were measured (Mettler Toledo XP6 Micro Balance) and mixed with 0.2-ml 0.9% sterile saline (NaCl) solution in 27-gauge insulin needles (catalog no. 10002–726, VWR). ACTH solution was injected (one time) into the ‘pectoralis major’ muscle at a dosage of 50-IU/kg body weight (Washburn *et al.*, 2003; Jankowski *et al.*, 2009). Injections occurred during the first sample collection period of the treatment (0900 hours).

Many experiments that have employed a biological stressor are not necessarily biologically significant or akin to events individuals would encounter in the wild, such as the use of saline injections to elicit a stress response (Dehnhard *et al.*, 2003; Washburn *et al.*, 2003). Biological stressors should be biologically significant, as investigators want to determine how the stress response reacts to natural stressors for field deployment. We chose a pointing bird dog as the biological stressor. Pointing bird dogs are used during bobwhite hunts and therefore represent a realistic stressor bobwhite may encounter (Palmer *et al.*, 2002; Mecozzi and Guthery, 2007). The leashed dog was brought into each aviary and placed near each individual pen where it proceeded to sniff and point at the bird for a total of 2 minutes per pen to elicit a stress response. Exposure to the dog occurred during the first sample collection period for the treatment (0900 hours) and 48 hours after the ACTH injection.

### Sample Preparation and Extraction

Sample preparation occurred at The University of Georgia. All samples from the study were freeze dried in a lyophilizer for a minimum of 24 hours to control fiber and water content. Samples were homogenized by finely grinding and mixing in a sterilized mortar and pestle. A total of 0.05 g of ground feces were mixed with 1 ml of 86% methanol for 30 minutes in a vortex mixer at 1800 rpm. Samples were then centrifuged for 20 minutes at 20 800 X *g* and 0.80 ml of the supernatant was removed. Extracted samples were not dried due to potential steroid loss from heating.

### Radioimmunoassay and Validation

We measured FCM levels in bobwhite fecal extracts using a standard commercially available corticosterone I^125^ radioimmunoassay kit (Cat. # 07120103, MP Biomedicals, Orangeburg, NY). In total, 577 bobwhite fecal samples were analysed in duplicate within 17 assays. Each assay included a mixture of individuals of different age, sex and treatments to account for inter-assay variation when interpreting results. We followed the MP Biomedicals protocol for the I^125^ corticosterone RIA included within the kit. Fecal samples used were not diluted with assay buffer and were vortexed shortly prior to beginning the RIA to resuspend samples. Reagents were not halved per MP Biomedicals protocol. All assay samples denoted test tubes received 100 μl of extracted bobwhite fecal samples. We conducted standard assay validation including assessment of parallelism and intra/inter-assay variation to confirm that the assay accurately and precisely measured corticosterone metabolites in bobwhite fecal samples (Jeffcoate, 1981; Grotjan and Keel, 1996; O’Fegan, 2000). We tested for parallelism by comparing the curve formed using serial dilutions of three individual fecal samples with the curve formed with the standard curve from the kit. To do the comparison, we used log-logit transformation of percentage binding for both our standard curve and serially diluted samples and plotted those transformed values against log-transformed dosages for the curve and the dilutions. We then statistically tested for homogeneity of slope among regression lines using procedures outline in Sokal and Rohlf (1995). The slopes of the four regression lines did not differ from one another (*F*_3,12_ = 1.046, *P* = 0.41) (see Figure S1 for data), indicating acceptable specificity when using this assay with extracted fecal samples that were collected from bobwhite. Inter-assay variation was determined to be 11.7% and intra-assay variation was determined to be 9.8%.

### Statistical Analysis

We used a Bayesian hierarchical modeling approach within the jagsUI package (Kellner, 2018) of R (R Core Team, 2017) to estimate in the effects of age, sex, treatment, weight and time-since-treatment initiation using the model described below. We parameterized the model such that each bird had its own intercept and slope (i.e. random intercepts and slopes) to properly account for individual variation in reference and treatment effects (Coppes *et al*., 2018). We assumed normal distributions for the random effects with a mean of 0 and vague gamma-distributed precision terms (1/variance). We used vague normal priors for the fixed effects with a mean of 0 with small precision (0.001). These priors were chosen because we had no prior information to justify the use of informative priors. The model was parameterized using the ‘effects’ parameterization where the fixed effects represented the difference from reference as follows:

$$\begin{align*} {\mu}_i=&\ {\beta}_{j,k, Reference}+{\beta}_{j,k, ACTH}\ast{X}_{ACTH}\\&+{\beta}_{j,k, Biological}\ast{X}_{Biological}+{\beta}_{j,k, TIME}\ast{X}_{TIME}\\&+{\beta}_{j,k, ACTH\ast TIME}\ast{X}_{ACTH\ast TIME}\\&+{\beta}_{j,k, Biological\ast TIME}\ast{X}_{Biological\ast TIME}\\&+{\beta}_{j,k, WEIGHT}\ast{X}_{WEIGHT} \end{align*}$$

where ${Cort}_i\sim Norm\Big({\mu}_i,\tau \Big)$ is the model likelihood and

$$ \tau \sim Gamma\left(0.1,0.1\right); $$

$$ {\beta}_k\sim Norm\left({\mu}_k,{\tau}_k\right); $$

$$ {\mu}_k\sim Norm\left(0,0.001\right); $$

$$ {\tau}_k\sim Gamma\left(0.1,0.1\right) $$

are model priors. The model was fitted for *i* = 1, 2, …, N where N represents the total number of observations, while *j* = 1, 2,…*n* denotes the number of individuals, and *k = 1, 2,…* denotes the number of fixed effects. Here *Cort* represents the FCM concentration for individual *j*. *β_Reference_* represents the intercept for each individual *j* during the control treatment. *β_ACTH_* represents the effect of ACTH each individual. *β_Dog_* characterizes the effect of the biological treatment. *β_Time_* symbolizes the change FCM during each treatment period. β_ACTH*Time_ denotes the interaction of the ACTH treatment and time. β_Dog*Time_ represents the interaction of the biological treatment and time*. β_Weight_* characterizes the effect in regard to weight for every unit increase in weight (grams). *X* represents the response variable for each respective fixed effect noted.

**Figure 1:**
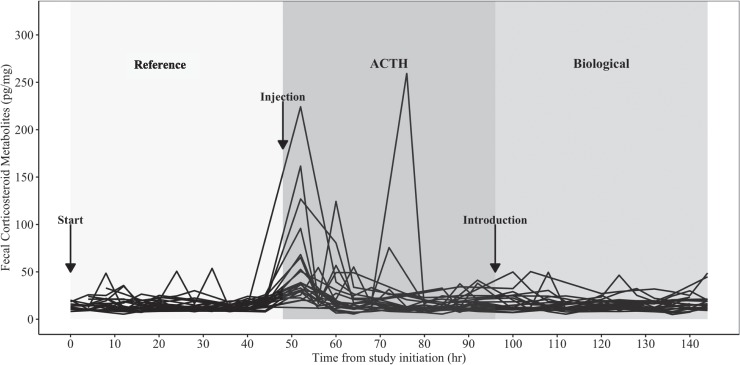
Observed FCM concentrations (pg/mg) of all Northern Bobwhite individuals (each line represents an individual) throughout the duration of the study. Samples were collected every 4 hours.

We used Markov chain Monte Carlo (MCMC) to estimate the posterior distributions of the model parameters. We generated three MCMC chains with 10 000 iterations, a burn in of 5000 and a thinning rate of one. This combination of values ensured an adequate number of iterations to characterize the posterior distributions, that MCMC chains showed no indications of autocorrelation or effects of initial values and that all chains converged. We checked chain convergence using the Gelman–Rubin statistic, R-hat, which compared between and within chain variation (Gelman *et al.*, 2004). R-hat values below 1.1 indicate convergence. Values of all estimated parameters were below 1.1. We report posterior means and 95% credible intervals. The degree of posterior overlap was used to determine biological significance. A *post hoc* test was conducted to check for a relationship between an individual’s response to ACTH and the biological stressor (i.e. negative feedback) through comparison of peak FCM concentrations of each individual in both treatments.

## Results

We collected and assayed an average of 25.2 ± 0.25 (SD) samples per bird (range, 5–34; *n* = 23) during the study (total of 577 samples) over a 144-hour collection period. Two mortalities occurred during the duration of the study, one within the habitation period and the other shortly after the initiation of the reference period. Of the 23 birds analysed, there were six adult males, six adult females, six juvenile males and five juvenile females. Bird weight was an average of 177 grams (range, 141–214 g).

**Figure 2:**
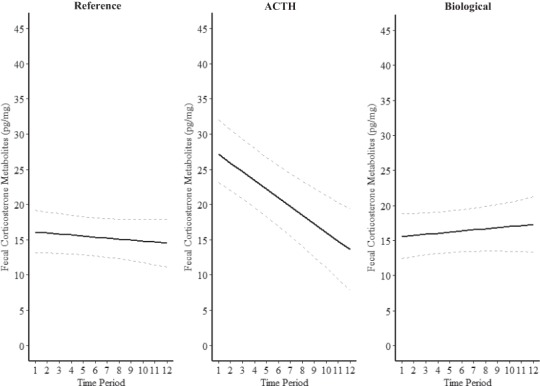
Model predicted values (solid lines) and 95% credibility intervals (dashed lines) of FCM concentrations (pg/mg) in Northern Bobwhite across time throughout the different study treatment.

Concentrations of FCM were relatively constant during the duration of the reference period with a slight decrease during the final collection time periods (Fig. 1, Fig. 2), resulting in average basal FCM concentrations of 16.75 pg/mg (Table 1). FCM concentrations decreased 12% to 14.68 pg/mg during the night, indicating a diurnal rhythm (Table 2). Individual variation within the reference period was 3.33 pg/mg (Table 2).

There was a pronounced increase in FCM concentrations during the ACTH treatment, with the peak concentration occurring during the first collection time period 4 hours after the injection occurred (Fig. 1, Fig. 2). FCM levels increased from 16.75 to 29.00 pg/mg (Table 2.1), resulting in a 73% increase on average in FCM concentrations during the ACTH period compared to reference. Concentrations returned to reference levels (16.75 pg/mg) during the final three collection time points of the treatment, approximately 38–44 hours after ACTH injection (Fig. 1, Fig. 2). Individual variation to the ACTH treatment was 1.23 pg/mg (Table 2). In contrast, FCM concentrations remained similar to that of the reference period (16.75 pg/mg) during the biological stressor treatment with an average concentration of 16.56 pg/mg (Table 1). Individual variation to the biological stressor treatment was 0.64 pg/mg (Table 2). Observed individual behavioral responses to the biological stressor ranged from producing alarm calls and running from the stressor to utilizing a common bobwhite anti-predator response known as holding, which allows individuals to remain cryptic within their landscape (Stoddard, 1931).

**Table 1:** Credible intervals and mean fecal corticosterone (pg/mg) concentrations of Northern Bobwhite for all posterior means

Parameters	0.025	0.25	Mean	0.75	0.975
Reference	13.68	15.68	16.75	17.80	19.91
ACTH	25.01	27.51	29.00	30.36	33.78
Biological	13.33	15.43	16.56	17.66	19.92

**Table 2:** Posterior means, credible intervals and individual variation (**σ)** for Northern Bobwhite fecal corticosterone (pg/mg) concentrations for all model variables

Parameters	0.025	0.25	Mean	0.75	0.975	σ
Reference	13.68	15.68	16.75	17.80	19.91	3.33
ACTH	8.49	10.83	12.26	13.48	17.23	1.23
Biological	−2.93	−1.21	−0.19	0.78	2.83	0.64
Time	−0.33	−0.12	−0.01	0.09	0.28	0.26
ACTH^*^time	−1.68	−1.30	−1.13	−0.96	−0.63	0.40
Biological^*^time	−0.24	0.03	0.17	0.31	0.56	0.26
Effect of night	−3.34	−2.51	−2.07	−1.64	−0.79	0.45
Weight	−4.64	−1.98	−1.06	0.03	1.95	0.77

The *post hoc* test to check for a relationship between an individual’s response to the ACTH challenge and the biological stressor showed that the ACTH challenge did not affect the results of the biological stressor, as there was a weak positive relationship between peak concentrations of both treatments observed. There was a weak positive relationship of 0.21 pg/mg (95% CrI 0.02, 0.42) between peak concentrations of the ACTH and biological treatments. Overall, there were no differences in FCM concentrations between sex and age classes or weight.

## Discussion

Our study indicated that FCM quantities in captive reared bobwhite reflect HPA activity in response to a pharmacological challenge. All sampled individuals responded to the ACTH injection, as indicated by a significant increase in FCM concentrations 4 hours post-injection and returning to reference levels 38–44 hours later. Concentrations of FCM remained relatively stable during the duration of the reference period and indicated no biologically relevant changes during the biological treatment. A weak diurnal pattern was detected with FCM concentrations being lower during the night.

Diurnal variations in glucocorticoids have been well documented in a number of vertebrate species, with peak concentration times differing between taxa (Halberg *et al*., 1959; Coe and Levine, 1995; De Jong *et al*., 2001). Variations tend to follow normal circadian rhythms, with higher concentrations occurring prior to daily activity with a continuous slow decline throughout the day (Romero, 2002). Our observation of a slight decrease in FCM concentrations at night follow this theory and are similar to that of domestic chickens (*Gallus gallus domesticus*), which were also found to have lower corticosterone concentrations at night (De Jong *et al*., 2001). The lack of a strong diurnal pattern may be due to the size of the samples extracted from the bobwhite. Sheriff *et al.* (2009a) found that the diurnal rhythm in snowshoe hares (*Lepus americanus*) was not detectable at a per gram scale, but rather at a total sample scale. Therefore, our extracted samples may have been too small to note a strong relationship. Understanding diurnal variations in species FCM levels is critical for interpreting results; therefore, collecting samples at the same time of day will bypass the complications of incorporating daily variations into analyses and interpretation (Touma and Palme, 2005; Dantzer *et al.*, 2014).

The stable FCM concentrations observed within the reference period possibly suggests that individuals had become acclimated to the presence of the investigators throughout the reference and habituation periods, as their presence did not cause any notable elevated stress responses. However, it could also mean that mild acute stress responses were not detectable via FCM. The reference levels detected in bobwhite were similar to that established in Japanese quail (*Coturnix japonica*), which were found to be 14.80 pg/mg (Hazard *et al.*, 2008). This response may be a result of the bobwhite being captive reared, as well as the fact that, in other birds, as time in captivity increases, basal corticosterone levels decrease (Wingfield *et al.*, 1982). This decrease in corticosterone levels is likely due to acclimation, whereas individuals cease to consider a stressor to be noxious after repeated chronic exposure (Romero, 2004). The stable concentrations throughout the reference period determined the average basal FCM level to which we could compare the effect of the latter treatments.

Increases in FCM concentrations following the injection of 50-IU/kg ACTH indicate that we were able to successfully induce changes in glucocorticoids within bobwhite and that our results reflect HPA activity to a pharmaceutical challenge. Detecting such changes in HPA activity is critical when validating FCM in a species, as it indicates that fluctuations in blood glucocorticoids were able to be successfully detected and monitored after metabolization, supporting the use of non-invasive FCM sampling (Palme, 2005; Palme, 2018). Similar ACTH peak response times of 4 hours have been noted in Mourning Doves (*Zenaida macroura*) (2–4 hours) and Greater Sage Grouse (*Centrocercus urophasianus*) (2–3 hours) post-injection (Washburn *et al.*, 2003; Jankowski *et al.*, 2009). Peak concentration times can differ between species and taxa due to gut passage times, with individuals such as snowshoe hares (*Lepus americanus*) reflecting peak ACTH concentrations 10 hours post-injection (Sheriff *et al.* 2009a).

In contrast to the ACTH treatment, FCM levels did not increase after individual exposure to the pointer dog. This lack of response was not due to the ACTH test inhibiting the results of the biological stressor, as there was a weak positive relationship between peak concentrations of the ACTH and biological treatment. This lack of elevation from reference FCM concentrations may be because the bobwhite were F1 captive reared birds and therefore did not have same ‘fight or flight’ responses when faced with a potential predator as a wild free-living bobwhite would due to acclimation (Wingfield *et al.*, 1982). Additionally, bobwhite (particularly captive reared) may not recognize dogs as a salient selection pressure and thus may not have the predicted glucocorticoid response. The absence of an elevation may further be due to the fact that bobwhite have not been exposed to pointer dogs as stressors over a long enough evolutionary time period to develop anti-predator responses to the animal. Furthermore, FCM concentrations are less affected by episodic fluctuations and therefore may not be as stimulated by a single event with a mild acute stressor such as a dog (Touma and Palme, 2005). However, Jones *et al.* (2016) did find that wild-caught European starlings (*Sturnus vulgaris*) had a profound adrenocortical stress response to only a short (<2–8 second) raptor attack, which would lead us to postulate that a 2-minute interaction with a pointer dog would lead similar responses. Future studies should expose the dog to the bobwhite multiple times throughout the study and for longer durations to attempt to elicit a detectable FCM response. We posit that a longer interaction will elevate FCM levels, as we did observe behavioral responses to the dog by the bobwhite via alarm calls, increased movement or attempting to remain cryptic within their holding pens. Additionally, the use of alternative biologically significant stressors such as anthropogenic hunting disturbance (e.g. a gunshot) or predator visibility may also be used in conjunction to elicit a detectable FCM stress response. Determination of the intensity of a biological stressor to cause a chronic effect that would be detectable in FCM is necessary to fully determine biological relevance of the method to understand how different FCM concentrations may affect individual fitness.

While the biological stressor may simply not be detectable via FCM due to it not chronically stressing the individuals, further validation is needed. Blood plasma samples would clarify if the biological treatment simply did not elicit a stress response from the individuals, or if FCM is insufficiently sensitive to detect a stress response that was short in duration. Additionally, the use of leukocyte profiles through white blood cell sampling may be useful as the results of this method are not affected by handling time and also allows for examination of an individual’s immune health (Davis *et al*., 2008). This study highlights that researchers cannot solely rely on the results of an ACTH challenge to indicate the biological relevance of FCM and the need for researchers to investigate how biological stressors of a variety of durations and magnitudes influence fecal FCM levels in order to fully understand how FCM may affect individual fitness. Moreover, this study reiterates the importance of validating the use of FCM in each species prior to large-scale field deployment of a sampling method.

As bobwhite are a species of conservation priority, FCM will be an important tool for wildlife managers and conservation biologists to assess the health of wild bobwhite and their populations. The use of FCM for bobwhite will have the most utility as a group- (i.e. covey) and population-level assay to monitor populations through time and compare chronic stress across populations to aid in the conservation of the species.

## Funding

This work was supported by the Georgia Department of Natural Resources and the Wildlife Restoration Program [grant number W-GA-F16AF00189].
